# Alvespimycin Exhibits Potential Anti-TGF-β Signaling in the Setting of a Proteasome Activator in Rats with Bleomycin-Induced Pulmonary Fibrosis: A Promising Novel Approach

**DOI:** 10.3390/ph16081123

**Published:** 2023-08-09

**Authors:** Osama A. Mohammed, Mustafa Ahmed Abdel-Reheim, Lobna A. Saleh, Mohannad Mohammad S. Alamri, Jaber Alfaifi, Masoud I. E. Adam, Alshaimaa A. Farrag, AbdulElah Al Jarallah AlQahtani, Waad Fuad BinAfif, Abdullah A. Hashish, Sameh Abdel-Ghany, Elsayed A. Elmorsy, Hend S. El-wakeel, Ahmed S. Doghish, Rabab S. Hamad, Sameh Saber

**Affiliations:** 1 Department of Clinical Pharmacology, College of Medicine, University of Bisha, Bisha 61922, Saudi Arabia; 2 Department of Pharmaceutical Sciences, College of Pharmacy, Shaqra University, Shaqra 11961, Saudi Arabia; 3 Department of Pharmacology and Toxicology, Faculty of Pharmacy, Beni-Suef University, Beni Suef 62521, Egypt; 4 Department of Clinical Pharmacology, Faculty of Medicine, Ain Shams University, Cairo 11566, Egypt; lobna_saleh@med.asu.edu.eg; 5 Department of Pharmacology and Toxicology, College of Pharmacy, Taif University, Taif 21944, Saudi Arabia; 6 Department of Family Medicine, College of Medicine, University of Bisha, Bisha 61922, Saudi Arabia; malamri@ub.edu.sa; 7 Department of Child Health, College of Medicine, University of Bisha, Bisha 61922, Saudi Arabia; jalfaifi@ub.edu.sa; 8 Department of Medical Education and Internal Medicine, College of Medicine, University of Bisha, Bisha 61922, Saudi Arabia; mieadam@ub.edu.sa; 9 Department of Histology and Cell Biology, Faculty of Medicine, Assiut University, Assiut 71515, Egypt; alshaima@aun.edu.eg; 10 Unit of Anatomy, Department of Basic Medical Sciences, College of Medicine, University of Bisha, Bisha 61922, Saudi Arabia; 11 Department of Internal Medicine, Division of Dermatology, College of Medicine, University of Bisha, Bisha 61922, Saudi Arabia; aaljarallah@ub.edu.sa; 12 Department of Internal Medicine, College of Medicine, University of Bisha, Bisha 61922, Saudi Arabia; waaeda@ub.edu.sa; 13 Department of Basic Medical Sciences, College of Medicine, University of Bisha, Bisha 61922, Saudi Arabia; ahsahish@ub.edu.sa; 14 Department of Clinical Pathology, Faculty of Medicine, Suez Canal University, Ismailia 41522, Egypt; 15 Department of Clinical Pharmacology, Faculty of Medicine, Mansoura University, Mansoura 35516, Egypt; samghany@mans.edu.eg (S.A.-G.); elsayedcp@mans.edu.eg (E.A.E.); 16 Pharmacology and Therapeutics Department, Qassim College of Medicine, Qassim University, Buraydah 51452, Saudi Arabia; 17 Physiology Department, Benha Faculty of Medicine, Benha University, Benha 13518, Egypt; hend.elwakel@fmed.bu.edu.eg; 18 Physiology Department, Albaha Faculty of Medicine, Albaha University, Al Baha 65799, Saudi Arabia; 19 Department of Biochemistry, Faculty of Pharmacy, Badr University in Cairo, Cairo 11829, Egypt; ahmed.soliman2@buc.edu.eg; 20 Department of Biochemistry and Molecular Biology, Faculty of Pharmacy (Boys), Al-Azhar University, Cairo 11231, Egypt; 21 Biological Sciences Department, College of Science, King Faisal University, Al Ahsa 31982, Saudi Arabia; rhamad@kfu.edu.sa; 22 Central Laboratory, Theodor Bilharz Research Institute, Giza 12411, Egypt; 23 Department of Pharmacology, Faculty of Pharmacy, Delta University for Science and Technology, Gamasa 11152, Egypt

**Keywords:** alvespimycin (17-DMAG), oleuropein, bleomycin-induced pulmonary fibrosis, HSP90/TGF-β, TβR, proteasome

## Abstract

Idiopathic pulmonary fibrosis (IPF) is an irreversible and life-threatening lung disease of unknown etiology presenting only a few treatment options. TGF-β signaling orchestrates a cascade of events driving pulmonary fibrosis (PF). Notably, recent research has affirmed the augmentation of TGF-β receptor (TβR) signaling via HSP90 activation. HSP90, a molecular chaperone, adeptly stabilizes and folds TβRs, thus intricately regulating TGF-β1 signaling. Our investigation illuminated the impact of alvespimycin, an HSP90 inhibitor, on TGF-β-mediated transcriptional responses by inducing destabilization of TβRs. This outcome stems from the explicit interaction of TβR subtypes I and II with HSP90, where they are clients of this cellular chaperone. It is worth noting that regulation of proteasome-dependent degradation of TβRs is a critical standpoint in the termination of TGF-β signal transduction. Oleuropein, the principal bioactive compound found in *Olea europaea*, is acknowledged for its role as a proteasome activator. In this study, our aim was to explore the efficacy of a combined therapy involving oleuropein and alvespimycin for the treatment of PF. We employed a PF rat model that was induced by intratracheal bleomycin infusion. The application of this dual therapy yielded a noteworthy impediment to the undesired activation of TGF-β/mothers against decapentaplegic homologs 2 and 3 (SMAD2/3) signaling. Consequently, this novel combination showcased improvements in both lung tissue structure and function while also effectively restraining key fibrosis markers such as PDGF-BB, TIMP-1, ACTA2, col1a1, and hydroxyproline. On a mechanistic level, our findings unveiled that the antifibrotic impact of this combination therapy likely stemmed from the enhanced degradation of both TβRI and TβRII. In conclusion, the utilization of proteasomal activators in conjunction with HSP90 inhibitors ushers in a promising frontier for the management of PF.

## 1. Introduction

Idiopathic pulmonary fibrosis (IPF) is an irreversible, progressive, and usually life-threatening lung disease of unknown etiology, with high morbidity and mortality—the average life expectancy is 3–5 years after the disease course [1]. The histopathological features of the disease are subpleural fibrosis, subepithelial fibroblast foci, and microscopic honeycombing [2]. The identification of the exact pathogenic mechanisms continues to evolve. Strong evidence suggests that the activated lung epithelial cells secrete mediators, leading to massive fibroblast proliferation and then differentiation to highly active myofibroblasts (the key effectors of fibrogenesis). These fibroblasts deposit superfluous amounts of extracellular matrix (ECM) proteins, such as collagen I, collagen III, actin alpha 2 (ACTA2), fibronectin, hyaluronic acid, and proteoglycans, that may evoke mesenchymal-cell activation and lung remodeling, disrupting the lung architecture irreversibly [3,4,5]. In the meantime, there are few treatment options for IPF treatment [6].

Epithelial cells are also thought to be the primary drivers of fibrogenesis because they can trans-differentiate and acquire a myofibroblastic phenotype, contributing to the accumulation of ECM in fibrotic tissue. This transformation, called the epithelial-to-mesenchymal transition (EMT), is operated by transforming growth factor beta 1 (TGF-β1) [7]. Transforming growth factor beta 1 is a crucial cytokine that is implicated in the process of fibrogenesis and can initiate the proliferation and transdifferentiation of myofibroblasts. It also enhances the formation of collagen, fibronectin, and other ingredients of the ECM by acting on TGF-β receptors (TβRs). Furthermore, TGF-β potentiates canonical TGF-β-SMAD signaling and drives EMT and resident fibroblast trans-differentiation [8]. 

There is strong evidence that heat shock protein (HSP) 90 plays a crucial role in the fibrogenesis process [9]. For instance, studies have proved that the TβR signaling pathway is driven by HSP90 activation in dermal fibrosis and renal fibrosis [10], but the impact of HSP90 in PF is largely unknown. Heat shock proteins are essential cellular chaperones, by this means facilitating the response to aggregated or misfolded proteins [11]. HSPs are a family of stress proteins whose expression is highly initiated by exaggerated temperature and would not be triggered under normal environmental conditions. Therefore, when living creatures are exposed to a series of physiological or environmental insults, such as ultraviolet radiation, heavy metals, membrane perturbations, or oxidative stress, HSPs are up-regulated and activated to support cell survival [12]. Otherwise, HSPs can promote the death of an irreparably damaged cell. This cytoprotective mechanism may be a result of consecutive pathways [13]. 

The HSPs family also provides ideal collagen conditions. They are collagen-specific molecular chaperones, whose presence stimulates the production of collagen [14]. The abnormal synthesis of massive amounts of collagen in tissues and organs is well known as fibrosis [15]. Therefore, HSPs have a critical role in fibrogenesis and fibrosis progression. Furthermore, HSP90 plays an essential role in IPF, as HSP90 is considered a molecular chaperone, which supports the folding and stabilization of TβRs; thus, HSP90 can regulate TGF-β1 signaling [16]. 

HSP90 inhibitors can efficiently prevent fibrotic diseases by inhibiting TGF-β1 signaling, EMT, and ECM production [17]. The inhibition of HSP90 has been found to mitigate fibrogenesis, such as skin fibrosis [18], myocardial fibrosis [19], renal fibrosis [20], and liver fibrosis [21]. On the other hand, HSP70 knocked-out mice exhibit elevated susceptibility to bleomycin (BLCN)-induced PF [22]. Deletion of HSP70 stimulates the signaling cascade of TGF-β activation, consequently increasing the production of ECM and exacerbating PF [23]. Additionally, in transgenic HSP70 mice, the BLCN-induced PF and inflammation are attenuated because the TGF-β signal was blocked. Another study demonstrated that the inhibition of HSP70 activates TGF-β and hence stimulates an in vitro EMT-like phenotype [22]. Moreover, it has been stated that the repression of HSP70 induction aggravates BLCN-mediated PF [24]. Further, Sellares, et al. [23] revealed that HSP70 gene deletion exhibits faster ECM deposition and eventually leads to significant fibrosis in mice. These data prove that HSP70 has protective effects on PF.

The HSP90 regulatory domain comprises an ATP-binding site. After the binding of ATP and its hydrolysis, the client/HSP90 complex integrates with co-chaperones to motivate the stabilization of the client protein. By contrast, HSP90, in its ADP-bound form, integrates with different cochaperones such as HSP70, leading to ubiquitin-mediated degradation of the client [25].

HSP90 inhibitors blocked TGF-β antiproliferative signaling. Regarding this effect, 17AAG-blocked HSP90 implicated the inhibition of TGF-β-mediated transcriptional responses by promoting TβR ubiquitination and degradation, thereby inhibiting SMAD2/3 signaling. In view of this, TβRI and TβRII are clients of HSP90 and interact particularly with this cellular chaperone [25]. This interaction accounts for the use of HSP90 inhibitors to counterbalance the unwanted activation of TGF-β signaling.

The first HSP90-specific inhibitor discovered was geldanamycin, which was isolated from the bacterium Streptomyces hygroscopicus. The inhibition of HSP90 by geldanamycin resulted in the degradation of its client proteins. However, geldanamycin is hydrophobic and causes toxicity in the liver and erythrocytes [26]. The HSP90 inhibitor alvespimycin, 17-DMAG, is less hepatotoxic and more water-soluble than geldanamycin. Therefore, in the present research, we aimed to investigate the protective effects of HSP90 inhibition using alvespimycin in an animal model of BLCN-induced PF and to explore the potential mechanisms thereof.

Lysosomes and proteasomes are the two major cellular proteolysis systems. While lysosomes are important for the decomposition of endocytosed proteins, proteasomes, or nonlysosomal threonine proteases, are the main protein degradation machinery in eukaryotes. Along with degrading damaged or misfolded proteins, evidence suggests that proteasomes are implicated in a diversity of critical cellular functions, including differentiation, cell cycle control, antigen processing in immune responses, stress signaling, inflammatory responses, apoptosis, and signal transduction [27]. Moreover, because many proteins undergo polyubiquitination before proteasomal degradation, the ubiquitin-proteasome cascade is critical for optimum cellular function [28]. Furthermore, it has been established that TβR1 and TβR2 can be degraded via ubiquitination-dependent or -independent degradation by the proteasome degradation system [29]. 

Oleuropein (OLPN) is the major phenolic compound in *Olea europaea* and traditionally is a main component of the Mediterranean diet. It has been found that OLPN leads to the stimulation of proteasome activities and can retain proteasome function in vitro [30]. Additionally, constant treatment of early passage human embryonic fibroblasts with OLPN reduced ROS intracellular levels and decreased the amount of oxidized proteins by promoting proteasome-mediated degradation rates [30]. Moreover, OLPN has been found to activate neonatal neocortical proteasomes in vivo [31]. Furthermore, OLPN has other several reported effects, including anti-inflammatory [32], antioxidant [33], anti-atherogenic [34], antimicrobial [35], anti-cancer [36], cardioprotective [37], neuroprotective [38], and hypolipidemic [39] activities. 

In the present study, we tested the assumption that HSP90 contributes to the development of PF. Additionally, we assessed the therapeutic effects of HSP90 inhibition by alvespimycin alone, or in combination with the known proteasome activator OLPN, in a bleomycin-induced PF rat model. It is worth noting that regulation of proteasome-dependent degradation of the receptors is an important aspect in the termination of TGF-β signal transduction. In this regard, the proteasome inhibits TGF-β signaling by interacting with TβRs and promotes their degradation [25]. Given this, we suggest that alvespimycin-mediated TGF-β receptor degradation may be enhanced by the proteasome activator OLPN.

## 2. Results

### 2.1. Effect of Alvespimycin and OLPN on Cell Viability

**Figure 1:**
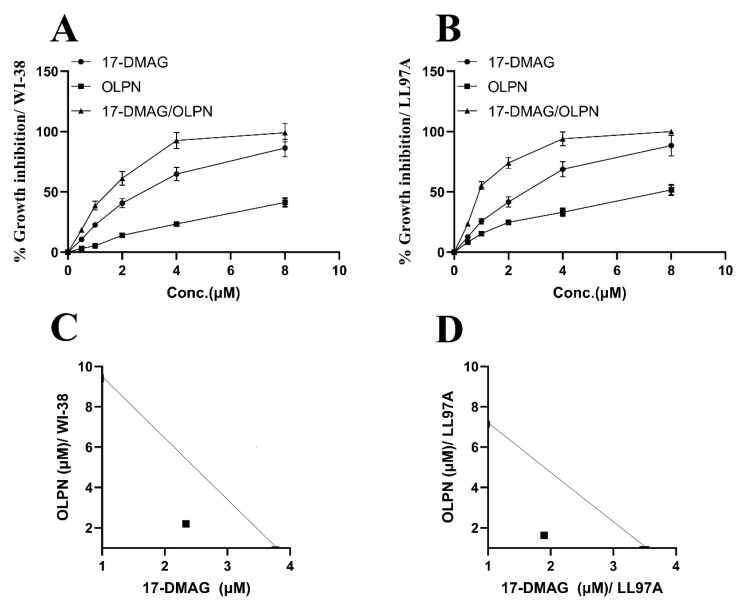
Effect of alvespimycin and OLPN on % growth inhibition in WI-38 fibroblast cell line (**A**), % growth inhibition in LL97A fibroblast cell line (**B**), combination index (WI-38) (**C**), and combination index (LL97A) (**D**). The growth inhibitory rates of the tested drugs were determined through linear regression analysis at various concentrations. In the WI-38 fibroblast cell line (**A**), the CTC50 values for alvespimycin and OLPN were 3.76 μM and 9.43 μM, respectively. Similarly, in the LL97A fibroblast cell line (**B**), the CTC50 values for alvespimycin and OLPN were 3.55 μM and 7.22 μM, respectively. Isobolograms were constructed to assess the combined effect of alvespimycin and OLPN in both cell lines. In (**C**) (WI-38), the experimental data points, along with their corresponding standard errors of the mean, were located beneath the theoretical additive point on the isobologram, revealing a synergistic effect between the two drugs at concentrations of 3.76 μM and 9.43 μM for alvespimycin and OLPN, respectively. Similarly, in (**D**) (LL97A), the experimental data points exhibited a synergistic interaction below the theoretical additive point at concentrations of 3.55 μM and 7.22 μM for alvespimycin and OLPN, respectively. This suggests that the combined effect of the drugs surpasses the individual effects.

The growth inhibitory effects of the drugs were evaluated at different concentrations using linear regression analysis. In the WI-38 fibroblast cell line (Figure 1A), the CTC_50_ values for alvespimycin and OLPN were determined to be 3.76 µM and 9.43 µM, respectively. Similarly, in the LL97A fibroblast cell line (Figure 1B), the CTC_50_ values for alvespimycin and OLPN were observed to be 3.55 µM and 7.22 µM, respectively.

### 2.2. Effect on the Combination Index between Alvespimycin and OLPN

Isobolograms were utilized to assess the combined impact of alvespimycin and OLPN in both the WI-38 and LL97A fibroblast cell lines. Figure 1C (WI-38) and Figure 1D (LL97A) illustrate that the experimental data points, along with their corresponding standard errors of the mean, were positioned beneath the theoretical additive point on the isobolograms. This finding indicates a synergistic effect between the two drugs at concentrations of 3.76 µM and 9.43 µM for alvespimycin and OLPN in the WI-38 cell line, respectively, and 3.55 µM and 7.22 µM for alvespimycin and OLPN in the LL97A cell line, respectively. In other words, the combined effect of the drugs exceeds what would be anticipated based solely on their discrete effects.

### 2.3. Histopathological Examination of Lung Tissue Sections

#### 2.3.1. H&E Stain

**Figure 2:**
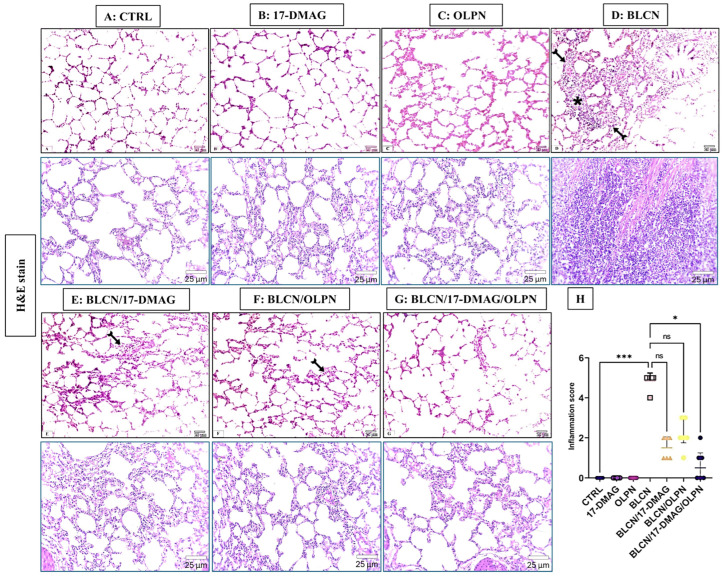
Effect of the alvespimycin, OLPN, and their combined therapy on the histological structure of the lung. Sections of CTRL (**A**), 17-DMAG (**B**), and OLPN (**C**), respectively, show normal lung architecture formed of thin-walled alveoli. Section of BLCN-treated rats (**D**) shows substantial inflammatory cellular infiltration (asterisk) and marked thickening of inter-alveolar septa (forked arrow) compared to CTRL, 17-DMAG, or OLPN rats. Lung sections from rats with pulmonary fibrosis treated with 17-DMAG (**E**), OLPN (**F**), and their combined therapy (**G**) reveal mild inflammatory cellular infiltration and alveolar septal thickening (forked arrows) as confirmed by the inflammation score (**H**). Significance is indicated by the pairwise comparisons. * *p* < 0.05 vs. BLCN; *** *p* < 0.001 vs. CTRL.

As depicted in Figure 2, compared to CTRL (A), 17-DMAG (B), and OLPN (C) rat groups, examination of H&E-stained sections from BLCN-treated rats showed distorted lung architecture with immense inflammatory cellular infiltration and marked thickening of inter-alveolar septa (D), which have normal lung architecture formed of alveoli separated by thin interalveolar septa. Lung sections from rats with pulmonary fibrosis that were treated with 17-DMAG (E), OLPN (F), and their combined therapy (G), revealed noticeable improvement and restoration of lung structure, compared to BLCN-treated rats. However, mild inflammatory cellular infiltration and occasional interalveolar septal thickening were observed. The score of inflammation data (H) confirmed the previous findings and revealed that the combination therapy significantly repealed the BLCN-induced elevation of the inflammation score.

#### 2.3.2. Masson’s Trichrome Stain

**Figure 3:**
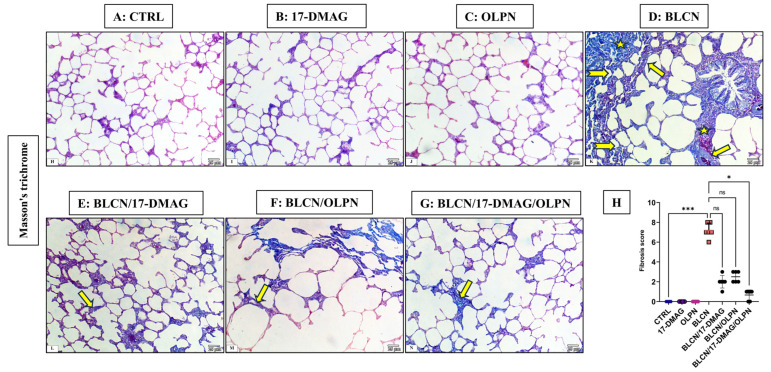
Masson’s trichrome stained sections of CTRL (**A**), 17-DMAG (**B**) and OLPN (**C**) respectively show fine collagen fibers in the interalveolar septa. Section of bleomycin-treated rats (**D**) shows increased collagen deposition (asterisks), marked septal thickening (arrows), and compressed alveoli (forked arrows). Sections of rats treated with 17-DMAG (**E**), OLPN (**F**), and their combined therapy (**G**) show mild deposition of collagen and mild septal thickening (arrow) in some areas and a significantly decreased percentage of lung fibrosis score (**H**) in lung sections. Significance is indicated by the pairwise comparisons. * *p* < 0.05; *** *p* < 0.001; ns: not significance.

As depicted in Figure 3, compared to CTRL (A), 17-DMAG (B), and OLPN (C) rat groups, Masson’s trichrome-stained sections of BLCN-treated rats showed excessive collagen deposition in the interalveolar septa and around the bronchioles, which resulted in septal thickening and compressed alveoli (D). In contrast, the collagen fiber deposition was markedly diminished in rats of the BLCN/17-DMAG (E), BLCN/OLPN (F), and their combined therapy (G) groups compared with the BLCN-treated rats. The ImageJ-calculated fibrosis area % data (H) confirmed these findings and revealed that the combination therapy significantly repealed the BLCN-induced elevation in the area % of fibrosis.

### 2.4. Effect on the Immune Expression of ACTA2 and p-SMAD2/3 

#### 2.4.1. Immuno-Expression of ACTA2

**Figure 4:**
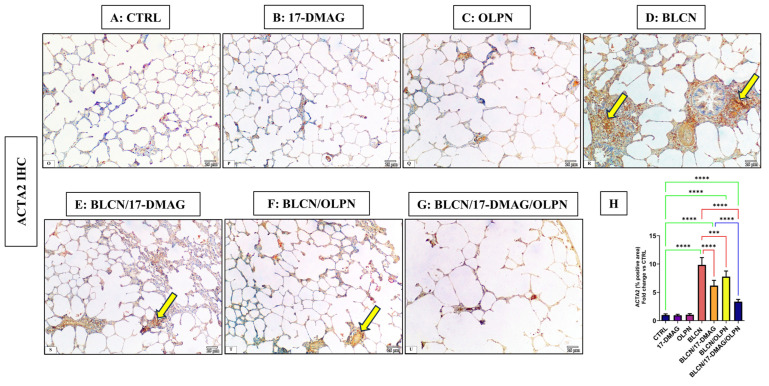
Regarding tissue expression of ACTA2, CTRL (**A**), 17-DMAG (**B**) and OLPN (**C**) show scant expressions in lung tissues. Sections of bleomycin-treated animals (**D**) show an increase in ACTA2 immune expression (arrows). Sections of rats with pulmonary fibrosis treated with 17-DMAG (**E**), OLPN (**F**), and their combined therapy (**G**) attained a significant reduction in tissue expression of these markers compared to bleomycin-treated animals as indicated by the improved percentage of the positive area (**H**). Significance is indicated by the pairwise comparisons. *** *p* < 0.001; **** *p* < 0.0001.

As depicted in Figure 4, compared to CTRL (A), 17-DMAG (B), and OLPN (C) rat groups, examination of ACTA2 stained sections of BLCN-treated rats showed a highly significant increase in the percentage of the positive area (D), whereas it was significantly lower in BLCN/17-DMAG (E) and BLCN/OLPN-treated (F) rats. There was a significant decrease in its expression in the combined treatment group (G). The ImageJ-calculated ACTA2 positive area data (H) confirmed these findings and revealed that the combination therapy significantly repealed the BLCN-induced elevation in the ACTA2 immunoexpression. 

#### 2.4.2. Immuno-Expression of p-SMAD2/3

**Figure 5:**
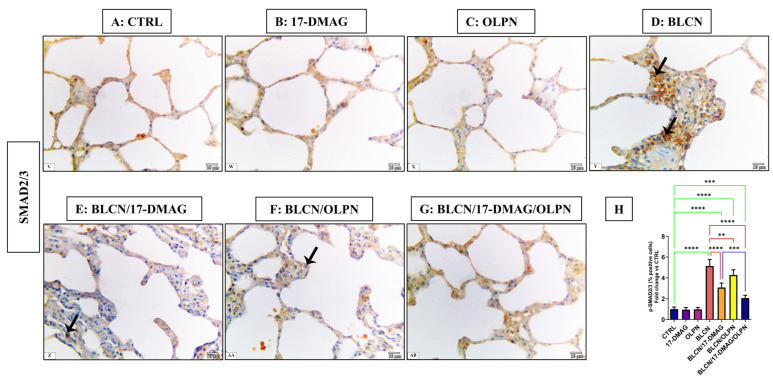
Regarding tissue expression of p-SMAD2/3, CTRL (**A**), 17-DMAG (**B**) and OLPN (**C**) show scant expressions in lung tissues. Sections of bleomycin-treated animals (**D**) show an increase in p-SMAD2/3 immune expression (arrows). Sections of rats with pulmonary fibrosis treated with 17-DMAG (**E**), OLPN (**F**), and their combined therapy (**G**) attained a significant reduction in tissue expression of p-SMAD2/3 compared to bleomycin-treated animals as also indicated by the decreased percentage of positive cells (**H**). Significance is indicated by the pairwise comparisons. ** *p* < 0.01; *** *p* < 0.001; **** *p* < 0.0001.

As depicted in Figure 5, compared to CTRL (A), 17-DMAG (B), and OLPN (C) rat groups, examination of p-SMAD2/3-stained sections of BLCN-treated rats showed a highly significant increase in the percentage of the positive cells (D), whereas it was significantly lower in BLCN/17-DMAG (E) and BLCN/OLPN-treated (F) rats. There was a significant decrease in the percentage of positive cells in the combined treatment group (G). The calculation of the percentage of the positive cells data (H) confirmed these findings and revealed that the combination therapy significantly repealed the BLCN-induced elevation in the percentage of the positive cells. 

### 2.5. Effect on Total Leukocyte Count, Lymphocyte Count, and Neutrophil Count in the BALF

**Figure 6:**
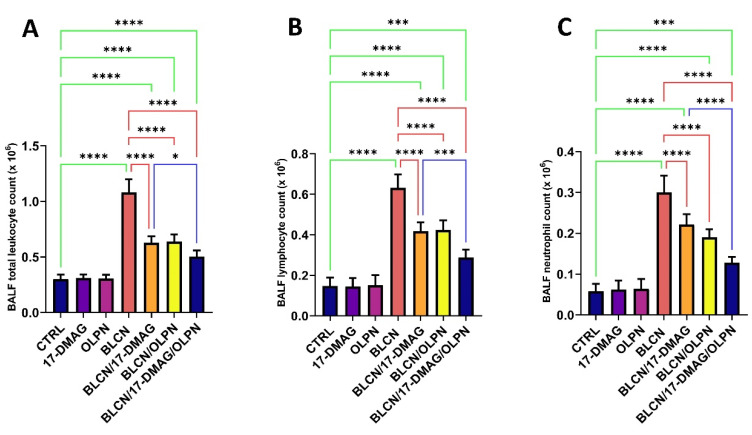
Effect of the combined therapy of alvespimycin and OLPN and their monotherapies on BALF total leukocyte count (**A**); lymphocyte count (**B**); and neutrophil count (**C**). Significance is indicated by the pairwise comparisons. * *p* < 0.05; *** *p* < 0.001; **** *p* < 0.0001.

Following BLCN administration, the total leukocyte count, lymphocyte count, and neutrophil count increased significantly in the BALF (Figure 6A,B,C, respectively). However, 17-DMAG, OLPN, or their combination treatment significantly reversed the observed effect caused by BLCN exposure. When compared to the BLCN/17-DMAG group, the combined therapy resulted in a significant decrease in these levels.

### 2.6. Effect on BALF Total Protein, LDH, and NOx

**Figure 7:**
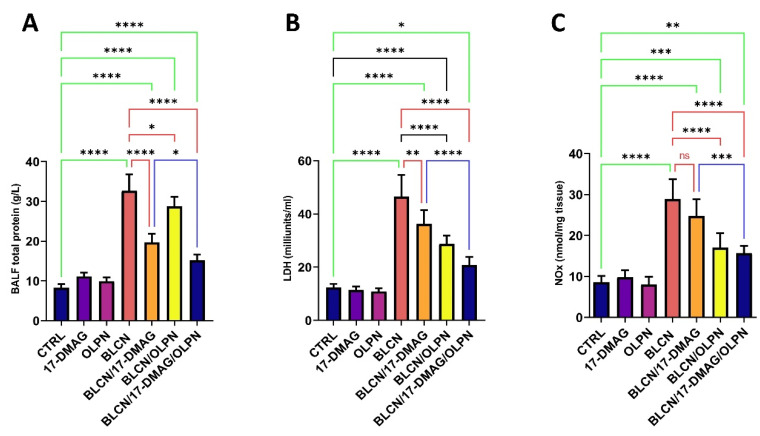
Effect of the combined therapy of alvespimycin and OLPN and their monotherapies on BALF total protein (**A**); LDH (**B**); and NOx (**C**). Significance is indicated by the pairwise comparisons. * *p* < 0.05; ** *p* < 0.01; *** *p* < 0.001; **** *p* < 0.0001.

The total protein content, LDH, and NOx levels in the BALF increased significantly after BLCN administration (Figure 7A,B,C, respectively). However, 17-DMAG, OLPN, or their combination treatment significantly reversed the BLCN-induced effect. The combined therapy led to a significant decrease in these levels in comparison with the BLCN/17-DMAG group.

### 2.7. Effect on MDA, SOD, and GSH

**Figure 8:**
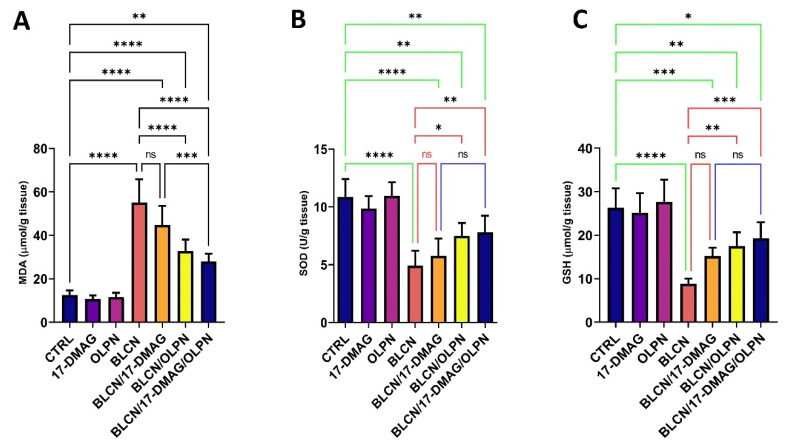
Effect of the combined therapy of alvespimycin and OLPN and their monotherapies on MDA (**A**); SOD (**B**); and GSH (**C**). Significance is indicated by the pairwise comparisons. * *p* < 0.05; ** *p* < 0.01; *** *p* < 0.001; **** *p* < 0.0001; ns: not significance.

We revealed that 17-DMAG as monotherapy did not result in a significant effect on the levels of oxidative stress markers MDA, SOD, and GSH, though a trend of significance is observed (Figure 8A,B,C, respectively). However, when combined with OLPN, 17-DMAG produces a significant effect on the levels of MDA compared to the BLCN and BLCN/17-DMAG groups. OLPN and, more significantly, the combination therapy, resulted in a rise in the levels of SOD and GSH compared to those of the BLCN rat group.

### 2.8. Effect on PDGF-BB, TIMP-1, COL1A1 mRNA, and Hydroxyproline

**Figure 9:**
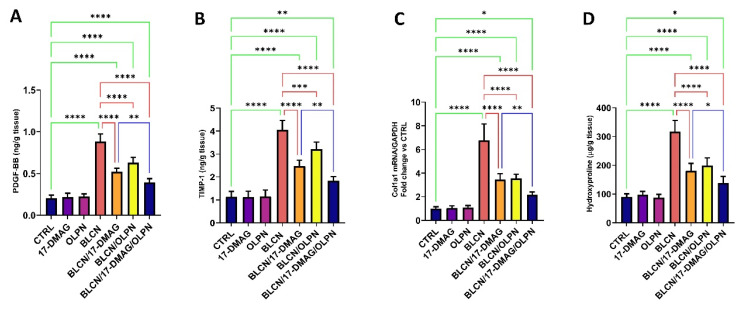
Effect of the combined therapy of alvespimycin and OLPN and their monotherapies on PDGF-BB (**A**); TIMP-1 (**B**); col1a1 (**C**); hydroxyproline (**D**). Significance is indicated by the pairwise comparisons. * *p* < 0.05; ** *p* < 0.01; *** *p* < 0.001; **** *p* < 0.0001.

17-DMAG or OLPN treatment to BLCN-exposed rats resulted in a significant rise in PDGF-BB, TIMP-1, COL1A1 mRNA, and hydroxyproline levels (Figure 9A,B,C,D, respectively) compared with the BLCN group which demonstrated a significant rise in these levels compared to those of the CTRL group. However, these levels were reduced significantly after the treatment of BLCN-exposed rats with 17-DMAG or OLPN. On the other hand, the combined therapy resulted in a significant decrease in these levels compared to those of the BLCN/17-DMAG group.

### 2.9. Effect on TGF-β mRNA, TGF-β, TβRI, and TβRII

**Figure 10:**
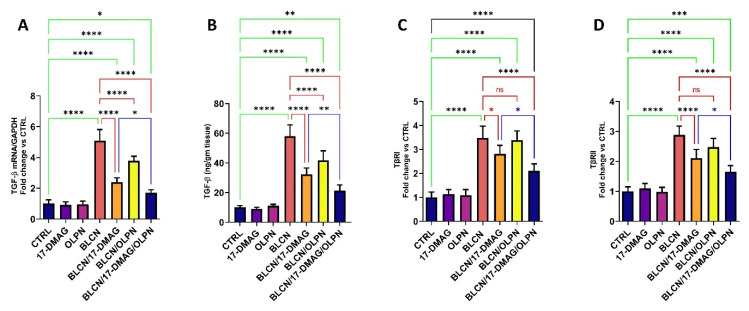
Effect of the combined therapy of alvespimycin and OLPN and their monotherapies on TGF-β mRNA (**A**); TGF-β (**B**); TβRI (**C**); and TβRII (**D**). Significance is indicated by the pairwise comparisons. * *p* < 0.05; ** *p* < 0.01; *** *p* < 0.001; **** *p* < 0.0001; ns: not significance.

The results of TGF-β mRNA and TGF-β (Figure 10A,B, respectively) were found to be parallel in that their levels were significantly elevated in the BLCN group compared to those of the CTRL group and found to be significantly reduced in both 17-DMAG and OLPN-treated pulmonary fibrosis rats. On the other hand, these levels were significantly decreased in the BLCN/17-DMAG/OLPN group compared to the BLCN/17-DMAG group. In addition, BLCN evoked an increase in the levels of both TβRI and TβRII (Figure 10C,D, respectively) compared to the CTRL group. However, these levels were significantly reduced compared to those of the BLCN group, especially the TβRII levels, after the treatment with 17-DMAG in rats with pulmonary fibrosis. In this regard, OLPN did not significantly alter the levels of both TβRI and TβRII. Interestingly, the combination of 17-DMAG with OLPN resulted in an extremely significant decrease in their levels compared to those of rats from the BLCN group. 

### 2.10. Effect of 17-DMAG and OLPN on 20S Proteasomal Activity in Lung Tissue

**Figure 11:**
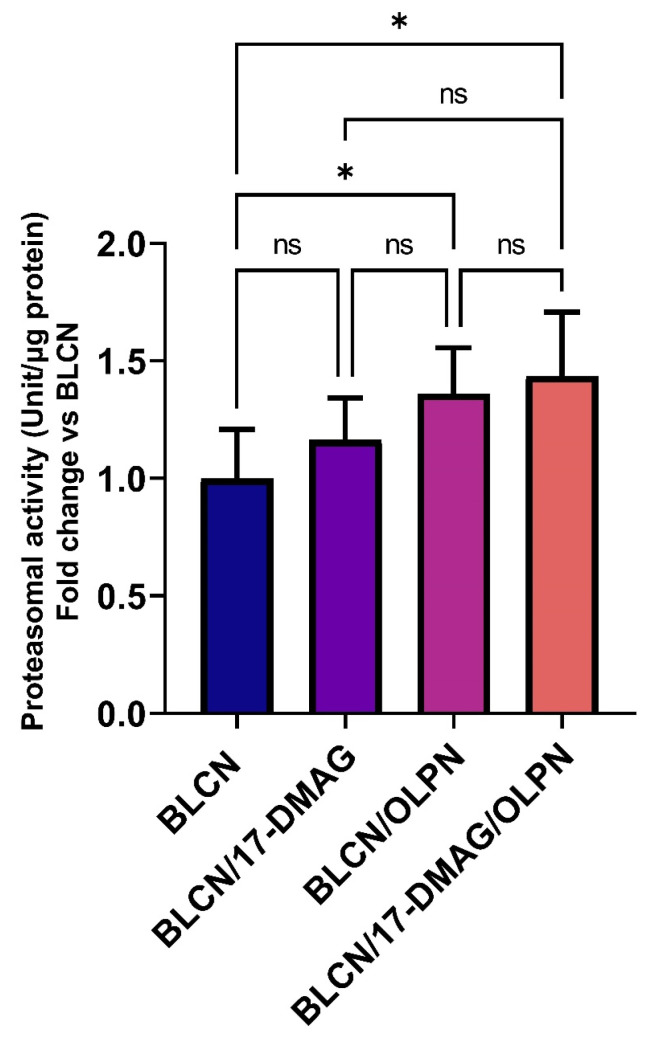
Effect of the combined therapy of alvespimycin and OLPN and their monotherapies on proteasomal activity. Significance is indicated by the pairwise comparisons. * *p* < 0.05; ns: not significance.

As illustrated in Figure 11, treatment with OLPN or 17-DMAG/OLPN led to a significant increase in 20S proteasomal activity in the lungs of rats exposed to BLCN when compared to the untreated BLCN group. This finding indicates that OLPN treatment, and not 17-DMAG, significantly enhanced 20S proteasomal activity in the fibrotic lungs of BLCN-exposed rats. The upregulation of this protein degradation pathway likely serves as one mechanism through which OLPN exerts its adjuvant effects by facilitating the breakdown of profibrotic proteins such as TβRs after being destabilized by HSP90 inhibition.

### 2.11. Effect on HSP70 and HSP90 

**Figure 12:**
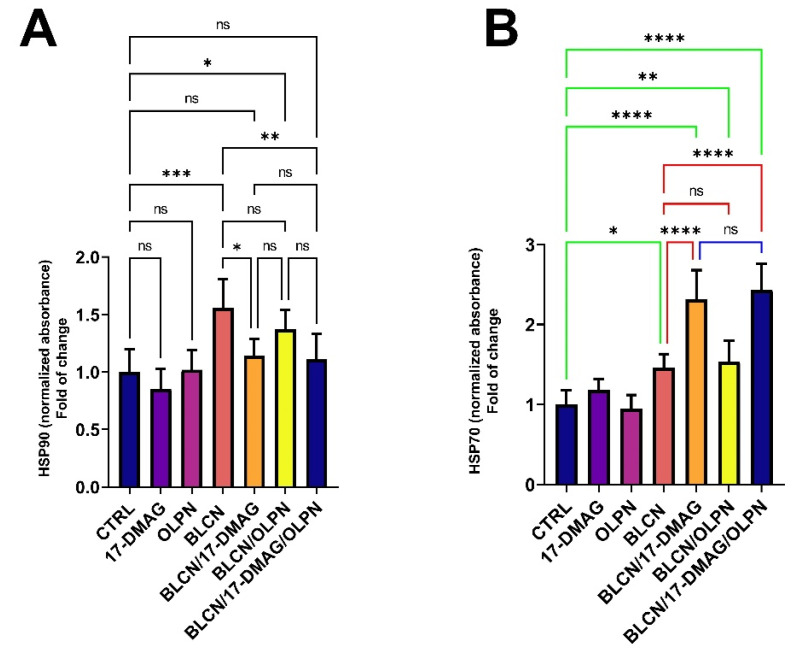
Effect of the combined therapy of alvespimycin and OLPN and their monotherapies on HSP90 (**A**) and HSP70 (**B**). Significance is indicated by the pairwise comparisons. * *p* < 0.05; ** *p* < 0.01; *** *p* < 0.001; **** *p* < 0.0001; ns: not significance.

We revealed that the levels of HSP90 (Figure 12A) were significantly increased in response to BLCN treatment. However, 17-DMAG and 17-DMAG/OLPN constrained this increase in the HSP90 levels. In parallel, BLCN treatment induced a rise in the levels of HSP70 (Figure 12B) compared to that of the CTRL group. However, HSP90 inhibition by 17-DMAG resulted in a significant increase in the levels of HSP70 in the BLCN/17-DMAG and BLCN/17-DMAG/OLPN groups compared to both the CTRL and BLCN groups. Further, we revealed that OLPN did not affect the HSP70 levels.

## 3. Discussion

Idiopathic pulmonary fibrosis (IPF) is a chronic, progressive lung disease that causes scar tissue (fibrosis) to build up in the lungs, making it difficult for the lungs to transport oxygen into the bloodstream effectively [40]. Currently, there is no cure for IPF, and lung damage from IPF is irreversible and progressive, meaning it gets worse over time. There is currently no cure for IPF, but medications such as nintedanib and pirfenidone can help slow down the progression of the disease and improve lung function [41].

TGF-β/SMAD signaling regulates interstitial lung fibroblast activation and extracellular matrix production, which is a pathological hallmark of IPF [42]. TGF-β has been recognized as a significant pro-fibrotic cytokine engaged in ECM protein synthesis [43]. Heat shock proteins have become increasingly involved in fibrotic diseases in recent years. Particularly, HSP90 forms complexes with many cellular proteins and is involved in a wide range of cellular reactions, including protein stabilization and degradation [44]. HSP90 functions as a molecular chaperone, promoting the stabilization and folding of TβRs and thus regulating TGFβ-1 signaling [25]. Therefore, we suggest that HSP90 inhibitors can efficiently repress fibrosis by inhibiting TGF-β1 signaling activation, EMT, and ECM synthesis [17]. Evidently, HSP90 inhibition repressed fibrogenesis, including liver fibrosis [45] and renal fibrosis [46].

Our data show the ability of alvespimycin to inhibit TGF-β1/SMAD interactions, suggesting that this HSP90 inhibitor could negatively regulate TGF-β signaling by affecting the stability of TβRs, especially TβRII. These findings are consistent with Wrighton, et al. [25], who revealed that TGF-β controls a diverse set of cellular processes by activating TβRI and TβRII serine-threonine receptor kinases, identifying a crucial role for HSP90 in TGFβ signaling. The authors also revealed that the blocking of HSP90 function by 17AAG, an HSP90 inhibitor, inhibits TGF-β-induced signaling and transcriptional responses.

TGF-β signaling is commenced by the binding of TGF-β to its serine and threonine kinase receptors, TβRI and the TβRII receptors on the cell membrane. Ligand binding results in the formation of the receptor hetero complex, in which TβRII phosphorylates threonine and serine residues in TβRI’s TTSGSGSG motif, activating TβRI [47]. In spite of the fact that they are protein kinases, TβRs also serve as phosphorylation substrates to regulate their activity [48]. TβRII is constitutively active and can undergo auto-phosphorylation [29] that may contribute to the marked effect on TβRII degradation by an HSP90 inhibitor. HSP90 specifically interacts with TβRI and TβRII in vitro and in vivo, and loss of HSP90 function reduces TβR levels and blocks TGF-β-induced SMAD2/3 activation and transcription. This finding suggests that HSP90 regulates TGF-β signaling as an essential component for TβR stabilization [25].

In the current study, bleomycin upregulated the expression levels of TβRI and TβRII whilst the administration of alvespimycin rather than OLPN downregulated these levels. Taking advantage of OLPN as a proteasome activator, the combination showed a pronounced decrease in the expression of TβRs. In this regard, alvespimycin facilitated the destabilization and exposure of the receptors to ubiquitination-dependent or -independent degradation by the OLPN-activated proteasomes. Because of the proteasome’s importance in many cellular processes, proteasome activation or inhibition is expected to affect many essential cellular functions. As a result, the potential application of proteasome regulators could be interesting and significant in many areas, including apoptosis, stress, and gene expression regulation. Further, OLPN is a naturally occurring small molecule that has been shown to enhance all proteasome activities at low micromolar concentrations [49]. 

When combined with 17-DMAG, OLPN has been found to enhance the degradation of TβRs that have been destabilized by 17-DMAG, leading to the inhibition of TGF-β signaling. This effect is likely achieved through the ability of OLPN to activate the proteasome, a cellular complex responsible for protein degradation. The regulation of proteasome-dependent degradation of the receptors plays a crucial role in terminating the transduction of TGF-β signals. Furthermore, the antifibrotic effects of OLPN observed in this study can be attributed to the reduction in the levels of the profibrogenic TGF-β. It is worth mentioning that OLPN is well-known for its antioxidant activity and its ability to inactivate NF-κB, a transcription factor involved in inflammation and fibrosis [50,51]. These properties of OLPN may contribute to the decreased transcription of TGF-β and other fibrogenic mediators.

Canonical TGF-β signaling is mediated by SMAD2 and SMAD3, which are phosphorylated in their SXS motif by activated TβRI. As stated by Noh, et al. [46], activated TβRI phosphorylates SMAD2/3, which partner with SMAD4 and translocate to the nucleus where they ultimately regulate gene transcription. Additionally, SMAD3 plays a pathologic role in fibrosis [43,52]. It directly binds to DNA sequences that regulate a number of fibrogenic genes (collagens types I & III) and markers (ACTA2 and E-cadherin). Moreover, overexpression of SMAD3 inhibits MMP-1 activity in fibroblasts, and the absence of SMAD3 blocks EMT and attenuates fibrosis [53]. Principally, the TGF-β/SMAD signaling pathway plays a key role in fibrosis through the attraction and proliferation of fibroblasts and the induction of EMT [54]. In this regard, TGF-β induces TIMP-1 by activating SMAD3, thus inhibiting ECM degradation [55]. Our data revealed that the combined therapy of alvespimycin and OLPN significantly decreased the tissue expression of p-SMAD2/3 compared to alvespimycin monotherapy. This effect might be attributed to the decreased protein expression of TβRs and resulted in mitigating pulmonary fibrosis induced by bleomycin.

Heat shock protein 70 has been shown to play a crucial role in preventing protein aggregation by redirecting unfolded and misfolded proteins to the ubiquitin-proteasome degradation system [56]. In addition, HSP70 has an antagonistic effect on EMT, which contributes to the process of fibrosis [57]. Moreover, it has been established that HSP70 is an emerging target for treating cystic fibrosis and may have the potential to be druggable [58]. Intriguingly, HSP90 inhibition results in an increase in the expression of HSP70, and therefore, HSP70 is considered a surrogate marker for the inhibition of HSP90 [59]. Our findings revealed that alvespimycin as monotherapy or in combination significantly increased the levels of HSP70. This result can be explained based on the inhibition of HSP90 by the effect of alvespimycin. 

In this study, BLCN-induced pulmonary fibrosis was indicated after calculating the fibrosis score, immunohistochemical examination of the expression of ACTA2, the gene expression level of col1a1, hydroxyproline lung content, and the protein expression levels of TIMP-1 and PDGF-BB. These parameters were prominently improved by the combined therapy of alvespimycin and OLPN. This antifibrotic property of the combined therapy was further supported after assessing the inflammation score, differential cell account, LDH, NOx in the BALF, and histological examination. Additionally, alvespimycin/OLPN exhibited antioxidant activity as indicated by increased GSH, SOD, and reduced lipid peroxidation. These protective effects might be attributed to the HSP90 inhibition-induced disruption in the TβRs that is followed by OLPN-facilitated proteasome degradation of the receptors. Subsequently, canonical TGF-β/SMAD2/3 signaling was interrupted as indicated by the levels of mRNA and the protein expression of TGF-β along with decreased tissue expression of p-SMAD2/3. 

## 4. Materials and Methods

### 4.1. Cell Culture and Cell Viability

The well-defined and cultured human lung fibroblast lines WI-38 and LL97A were acquired from ATCC in Manassas, USA. They were then cultivated in high-glucose DMEM (Invitrogen, Carlsbad, CA, USA) supplemented with 10% FBS, 2 mM glutamine, 50 µg/mL streptomycin, and 50 U/mL penicillin. The cells were maintained at a temperature of 37 °C in a humidified environment with 5% CO_2_. WI-38 or LL97A cells were passaged and plated onto 96-well plates with a density of 5000 cells per well. After 24 h, the culture media were replaced with 100 μL of media containing 0.5% DMSO and various concentrations of alvespimycin (0, 1, 2, 4, and 8 µM) from Tocris Bioscience (Bristol, UK) or OLPN from Sigma-Aldrich (St. Louis, MO, USA) (0, 1, 2, 4, and 8 µM), or a combination using the same concentration range. The samples were then incubated for 48 h, followed by the addition of 20 μL of a 5 mg/mL solution of MTT to each well. The plates were further incubated for 4 h. Following three washes with PBS, the cells were treated with 200 μL of DMSO to dissolve the formazan crystals. The absorbance of the cells was then measured at a wavelength of 490 nm using a microplate reader (Bio-Rad Laboratories, Hercules, CA, USA). Triplicate assays were performed, and the percentage of growth inhibition was determined using a previously described method [55]. 

### 4.2. Determination of the Index of Combination 

To explore the alvespimycin and OLPN interaction in WI-38 and LL97A fibroblast cell lines, isobolograms were created. This involved positioning the CTC_50_ values and standard error of the mean for each particular drug on the x and y axes. The experimentally determined CTC_50_ value for the combination of both drugs was also plotted. Furthermore, the combination index was confirmed using the formula described by Saber, et al. [60].

### 4.3. Induction of Pulmonary Fibrosis in Rats 

The present work was conducted using male adult SD rats weighing 220 ± 15 g that were purchased from the animal facility at TBRI. They were maintained in standard environmental conditions and were handled per the Research Ethics Committee guidelines (approval number, FPDU2, 2023). Under a chemical hood, bleocel^®^ (Bad Vilbel, Germany) was prepared by dissolving in saline and was intratracheally infused at a dose of 5 mg/kg into rats that were anesthetized with an intraperitoneal injection of sodium thiopental at a dose of 40 mg/kg. Rats were lying on their dorsal side on a surgical platform and were removed from the midline neck hair. The trachea was then exposed by making a short incision succeeded by deliberately injecting the bleomycin solution. The wounded area was then disinfected with 1% povidone-iodine solution. The animals were kept warm while lying on their sides and closely monitored for indications of pain, respiratory distress, or any signs of illness, until they fully recovered.

### 4.4. Study Design

As described in Table 1, animals were indiscriminately allocated to 7 groups as follows: **CTRL** group (*n* = 6), representing normal control rats; **17-DMAG** (*n* = 6) and **OLPN** (*n* = 6) groups, representing drug control groups; **BLCN** group (*n* = 12), in which rats received endoatracheal bleomycin (BLCN) once at the start of the experimental protocol; **BLCN/17-DMAG** group (*n* = 12), rats received endoatracheal BLCN plus 17-DMAG (10 mg/kg/thrice a week, p.o./3 weeks); **BLCN/OLPN** group (*n* = 12), rats received endoatracheal BLCN plus OLPN (50 mg/kg/day, p.o./3 weeks); **BLCN/17-DMAG/OLPN** group (*n* = 12), rats received endoatracheal BLCN plus 17-DMAG and OLPN. Drug administration began on the next day of the surgery and lasted till the 21st day. This is followed by euthanizing the animals on the 22nd day by decapitation after anesthetization with Ketamine/Xylazine mixture [61].

### 4.5. Rationale of Drug Dosing 

Alvespimycin was recently used at a dosage of 10 mg/kg thrice a week, p.o. in mice with hepatocellular carcinoma [59]. Additionally, alvespimycin was used every two days in mice with BLCN-induced PF via oral gavage at a dosage of 10 or 25 mg/kg [62]. OLPN was previously administered to rats at a dose of 50 mg/kg by gavage for 8 weeks [63].

### 4.6. Histological Examination of Lung Tissue Sections

On the last day of the experiment, anesthetized animals were euthanized, then left lungs were dissected and cleaned with cold PBS and preserved in formalin for 24 h. This was followed by the embedding of the lung tissues in paraffin. Lung sections were then cut at 4–5 µm thickness with a rotary microtome followed by a standard H&E and Masson’s trichrome staining procedures as previously described [64]. The scoring system, to assess the inflammatory changes, was implemented as previously described [43,65]. Additionally, the characteristic features used for the grading of the fibrotic changes were adopted from a previous study [1]. 

### 4.7. Immunohistochemical Examination of Lung Tissue Sections

Rabbit ACTA2 polyclonal antibodies (1:800 dilution) from Thermo Fisher Scientific (Rockford, IL, USA) and rabbit p-SMAD2/3 polyclonal antibodies (1:400 dilution) from Abcam (Cambridge, MA, USA) were used for the immunostaining of ACTA2 and p SMAD2/3 in lung sections, respectively, using the peroxidase-labeled streptavidin-biotin technique. Lung sections were incubated for 30 min with the previously mentioned primary antibodies, then bathed several times with phosphate-buffered saline. Goat anti-rabbit secondary antibodies were applied to the sections for 30 min at room temperature, incubated with the avidin-biotin compound, then with peroxidase substrate solution for 5 min to mask the endogenous peroxide. Sections were visualized utilizing DAB and counter-stained with Mayer’s hematoxylin. To determine ACTA2 expression, the proportion of the positive area in relation to the overall area was evaluated in 10 different HPF using Imagej 1.53p. The expression of p-SMAD2/3 is reported as the percentage of positive cells observed in ten different HPF that were counted.

### 4.8. Determination of Bronchoalveolar Lavage Fluid (BALF) Total Leukocyte, Neutrophil, and Lymphocyte Count

After opening the thoracic cavity and exposing the lungs, the left bronchus was clamped. A 16-gauge cannula was then inserted into the trachea in its original position to gently introduce three milliliters of cooled PBS solution into the right lung, administering one milliliter at a time. Throughout the procedure, the right lungs were carefully compressed to ensure the efficient retrieval of the instilled solution. The BALF obtained, which accounted for 75% of the initial volume, was subjected to centrifugation for 10 min at 2000 rpm in a cooled centrifuge. The separated pellets were used to determine the total leukocyte count, neutrophil count, and lymphocyte count. 

### 4.9. Determination of Lactate Dehydrogenase (LDH) Activity, BALF Total Protein, and Total Nitrite and Nitrate (NOx)

The total protein content of BALF was determined using a bicinchoninic acid protein assay kit (Thermo Scientific, Waltham, MA, USA). Sigma-Aldrich provided the LDH enzyme activity assay kit. Total nitric oxide and n,itrate/nitrite parameter assay kit was used for the determination of NOx (R&D systems, Minneapolis, MN, USA) according to the manufacturer’s instructions.

### 4.10. Determination of Malondialdehyde (MDA), Superoxide Dismutase (SOD), and Reduced Glutathione (GSH)

Malondialdehyde levels in lung tissues were determined using a thiobarbituric acid reactive substances assay provided by Sigma-Aldrich. To measure superoxide dismutase (SOD) activity in lung tissues, the SOD assay kit from Cayman Chemical (Ann Arbor, MI, USA) was utilized. The levels of GSH were determined using a colorimetric assay kit from Biodiagnostic (Dokki, Giza, Egypt) per the manufacturer’s instructions.

### 4.11. Determination of Platelet-Derived Growth Factor BB (PDGF-BB), Tissue Inhibitor of Metalloproteinase (TIMP-1), and Hydroxyproline

The determination of PDGF-BB was performed using the fine Test kit from Wuhan Fine Biotech Corp. (Wuhan, China). The concentration of TIMP-1 in lung tissues was determined using a kit supplied by RayBiotech (Norcross, GA, USA). Hydroxyproline was measured spectrophotometrically based on oxidation reaction. The produced oxidation product reacts with dimethylaminobenzaldehyde and presents a dark reddish-purple color. The OD is measured at 550 nm and the concentration of hydroxyproline is determined knowing the wet weight of lung tissue, OD of blank, and OD of the standard. All procedures were performed in accordance with the provided instructions.

### 4.12. Determination of TGF-β mRNA and Collagen Type I, Alpha 1 (COL1A1) mRNA

The mRNA from lung samples was extracted, using an RNeasy mini kit, from the right lungs that had been preserved in RNA previously. cDNA was made by reversing mRNA (Qiagen), and qRT-PCR was performed in a thermocycler with SYBR Green PCR Master Mix (Qiagen). The comparative cycle threshold (Ct) (2^−ΔΔCT^) was used to calculate the relative expression of TGF-β mRNA and COL1A1 mRNA. Table 2 summarises the primer sequences.

### 4.13. Determination of TGF-β, TβRI, and TβRII

According to the manufacturer’s instructions, TGF-β protein expression was measured using a kit provided by MyBioSource Inc. (San Diego, CA, USA). TβRI and TβRII were measured by kits that were provided by Abbexa (Cambridge, UK).

### 4.14. Determination of HSP70 and HSP90

The levels of HSP70 and HSP90 were quantified using ELISA kits obtained from MyBioSource Inc. and Novus Biologicals (Littleton, CO, USA), respectively, according to the manufacturer’s instructions.

### 4.15. Determination of the Proteasomal Activity in Lung Tissue

The experimental groups included the BLCN group, BLCN/17-DMAG group, BLCN/OLPN group, and BLCN/17-DMAG/OLPN group. Isolated lungs weighing 10 mg each were homogenized using a dounce tissue homogenizer. Subsequently, 100 μL of 20S IP assay buffer was added, and the tissue was homogenized on ice for 5 min to facilitate tissue lysis. The resulting lysate was subjected to centrifugation at 10,000× *g* and 4 °C for 10 min, leading to the collection of the supernatant for further analysis. The sample volume was adjusted to 50 μL using a 20S IP assay buffer, and the sample was then added to two wells labeled as “sample” and “inhibitor”. In the “inhibitor” well, 1 μL of 20S IP inhibitor was added to specifically isolate the proteasome activity from other protease activities present in the samples. The volume was adjusted to reach a total of 50 μL using 20S IP assay buffer. In the substrate background control well, a volume of 50 μL of 20S IP assay buffer was utilized. In the positive control well, 2 μL of 20S IP positive control was added, and the volume was adjusted to 50 μL using 20S IP assay buffer. Fluorescence measurements were conducted at excitation and emission wavelengths of 350 nm and 440 nm, respectively, at a temperature of 37 °C. To generate the AMC (7-amino-4-methylcoumarin) standard curve, the reading from the zero standard was subtracted from all the standard readings. Then, the inhibitor reading was subtracted from all the sample readings to obtain the corrected sample readings. These corrected sample readings were then applied to the previously constructed AMC standard curve to determine the amount of AMC present in the samples, measured in picomoles (pmol). It is worth noting that one unit of 20S immunoproteasome refers to the quantity of the enzyme that catalyzes the production of 1.0 micromole (μmol) of AMC per minute at a temperature of 37 °C. The protein concentration of each sample was assessed using the BCA protein assay kit, and the resulting values were normalized by the protein concentration. The proteasome activity, represented as Unit/μg of total proteins ± SEM, was then normalized to the proteasome activity of the BLCN group, which served as the control. The data are presented as the fold change relative to the BLCN samples, which were assigned a value of 1. A total of six independent assays were conducted for each group, and each assay was performed in duplicate.

### 4.16. Statistical Analysis

The statistical differences between groups were tested using the GraphPad Prism 9.4.0 software. For parametric data, one-way ANOVA was used, followed by Tukey’s Kramer post hoc multiple comparisons tests, and for non-parametric data, the Kruskal–Wallis test was used, followed by Dunn’s post hoc test. Statistical significance was considered for *p* values below 0.05. The data is presented as either the mean ± standard deviation for parametric data or the median ± interquartile range for non-parametric data.

## 5. Conclusions

**Figure 13:**
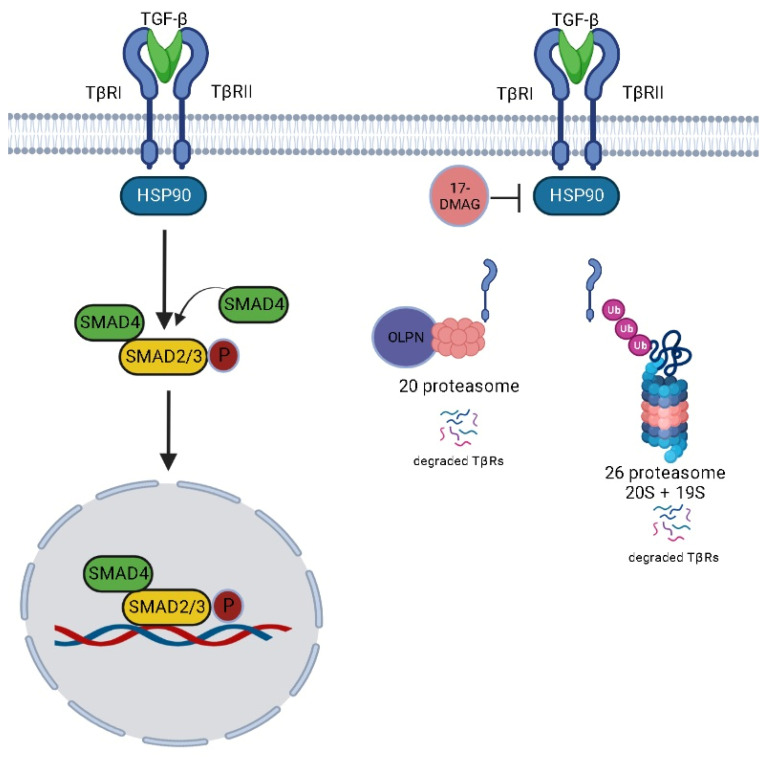
Proposed mechanism for the effect of alvespimycin and OLPN.

Our data reveal a critical level of TGF-β signaling regulation that is mediated by HSP90 via its ability to chaperone TβRs (Figure 13). Additionally, we point to the use of HSP90 inhibitors in blocking undesired activation of TGF-β signaling in pulmonary fibrosis. Moreover, we implicate the use of OLPN, the proteasome activator, as adjuvant therapy to alvespimycin to facilitate the proteasomal degradation of TβRs. To conclude, the use of proteasomal activators along with HSP90 inhibitors represents an innovative perspective in managing pulmonary fibrosis. However, further investigations are still ongoing in our laboratory. We highlight the importance of using immunohistochemical labeling of HSP90 and HSP70 as a confirmatory step to provide information about the localization in lung tissue during the progression of fibrosis.

## Figures and Tables

**Table 1:** Experimental design.

Exp. Groups	Day 1	Day 2–21
**CTRL** (*n* = 6)	Surgical operation Endotracheal saline	Vehicle
**17-DMAG** (*n* = 6)	Surgical operation Endotracheal saline	17-DMAG (10 mg/kg/thrice a week, p.o.)
**OLPN** (*n* = 6)	Surgical operation Endotracheal saline	OLPN (50 mg/kg/day, p.o.)
**BLCN** (*n* = 12)	Surgical procedure Endotracheal infusion of bleomycin (5 mg/kg)	-
**BLCN/17-DMAG** (*n* = 12)	Surgical operation Endotracheal infusion of bleomycin (5 mg/kg)	17-DMAG (10 mg/kg/thrice a week, p.o.)
**BLCN/OLPN** (*n* = 12)	Surgical operation Endotracheal infusion of bleomycin (5 mg/kg)	OLPN (50 mg/kg/day, p.o.)
**BLCN/17-DMAG/OLPN** (*n* = 12)	Surgical operation Endotracheal infusion of bleomycin (5 mg/kg)	17-DMAG (10 mg/kg/thrice a week, p.o.) + OLPN (50 mg/kg/day, p.o.)

BLCN, bleomycin; OLPN, oleuropein.

**Table 2:** Primer sequences.

Gene	GenBank Accession	Forward Primer	Reverse Primer	Amplicon Size (bp)
*Col1a1*	NM_053304.1	5′-GACATGTTCAGCTTTGTGGACCC-3′	5′-AGGGACCCTTAGGCCATTGTGTA-3′	120
*TGF-β1*	NM_021578.2	5′-CTTCTCCACCAACTACTGCTTC- 3′	5′-GGGTCCCAGGCAGAAGTT-3′	139
*GAPDH*	NM_001289726.1	5′-TCAAGAAGGTGGTGAAGCAG-3′	5′-AGGTGGAAGAATGGGAGTTG-3′	111

## Data Availability

Data is contained within the article.

## Abbreviations

ACTA2, actin alpha 2; BALF, bronchoalveolar lavage fluid; COL1A1, collagen type I, alpha 1; ECM, extracellular matrix; EMT, epithelial-to-mesenchymal transition; GSH, reduced glutathione; HSPs, Heat shock proteins; IPF, Idiopathic pulmonary fibrosis; LDH, lactate dehydrogenase; MDA, malondialdehyde; NOx, total nitrite and nitrate; OLPN, Oleuropein; PDGF-BB, platelet-derived growth factor-BB; SOD, superoxide dismutase; TGF-β, transforming growth factor beta 1; TIMP-1, tissue inhibitor of metalloproteinase; TβRs, TGF-β receptors.
