# Correction: Mohammed et al. Alvespimycin Exhibits Potential Anti-TGF-β Signaling in the Setting of a Proteasome Activator in Rats with Bleomycin-Induced Pulmonary Fibrosis: A Promising Novel Approach. *Pharmaceuticals* 2023, *16*, 1123

**DOI:** 10.3390/ph18071011

**Published:** 2025-07-07

**Authors:** Osama A. Mohammed, Mustafa Ahmed Abdel-Reheim, Lobna A. Saleh, Mohannad Mohammad S. Alamri, Jaber Alfaifi, Masoud I. E. Adam, Alshaimaa A. Farrag, AbdulElah Al Jarallah AlQahtani, Waad Fuad BinAfif, Abdullah A. Hashish, Sameh Abdel-Ghany, Elsayed A. Elmorsy, Hend S. El-wakeel, Ahmed S. Doghish, Rabab S. Hamad, Sameh Saber

**Affiliations:** 1 Department of Clinical Pharmacology, College of Medicine, University of Bisha, Bisha 61922, Saudi Arabia; 2 Department of Pharmaceutical Sciences, College of Pharmacy, Shaqra University, Shaqra 11961, Saudi Arabia; 3 Department of Pharmacology and Toxicology, Faculty of Pharmacy, Beni-Suef University, Beni Suef 62521, Egypt; 4 Department of Clinical Pharmacology, Faculty of Medicine, Ain Shams University, Cairo 11566, Egypt; lobna_saleh@med.asu.edu.eg; 5 Department of Pharmacology and Toxicology, College of Pharmacy, Taif University, Taif 21944, Saudi Arabia; 6 Department of Family Medicine, College of Medicine, University of Bisha, Bisha 61922, Saudi Arabia; malamri@ub.edu.sa; 7 Department of Child Health, College of Medicine, University of Bisha, Bisha 61922, Saudi Arabia; jalfaifi@ub.edu.sa; 8 Department of Medical Education and Internal Medicine, College of Medicine, University of Bisha, Bisha 61922, Saudi Arabia; mieadam@ub.edu.sa; 9 Department of Histology and Cell Biology, Faculty of Medicine, Assiut University, Assiut 71515, Egypt; alshaima@aun.edu.eg; 10 Unit of Anatomy, Department of Basic Medical Sciences, College of Medicine, University of Bisha, Bisha 61922, Saudi Arabia; 11 Department of Internal Medicine, Division of Dermatology, College of Medicine, University of Bisha, Bisha 61922, Saudi Arabia; aaljarallah@ub.edu.sa; 12 Department of Internal Medicine, College of Medicine, University of Bisha, Bisha 61922, Saudi Arabia; waaeda@ub.edu.sa; 13 Department of Basic Medical Sciences, College of Medicine, University of Bisha, Bisha 61922, Saudi Arabia; ahsahish@ub.edu.sa; 14 Department of Clinical Pathology, Faculty of Medicine, Suez Canal University, Ismailia 41522, Egypt; 15 Department of Clinical Pharmacology, Faculty of Medicine, Mansoura University, Mansoura 35516, Egypt; samghany@mans.edu.eg (S.A.-G.); elsayedcp@mans.edu.eg (E.A.E.); 16 Pharmacology and Therapeutics Department, Qassim College of Medicine, Qassim University, Buraydah 51452, Saudi Arabia; 17 Physiology Department, Benha Faculty of Medicine, Benha University, Benha 13518, Egypt; hend.elwakel@fmed.bu.edu.eg; 18 Physiology Department, Albaha Faculty of Medicine, Albaha University, Al Baha 65799, Saudi Arabia; 19 Department of Biochemistry, Faculty of Pharmacy, Badr University in Cairo, Cairo 11829, Egypt; ahmed.soliman2@buc.edu.eg; 20 Department of Biochemistry and Molecular Biology, Faculty of Pharmacy (Boys), Al-Azhar University, Cairo 11231, Egypt; 21 Biological Sciences Department, College of Science, King Faisal University, Al Ahsa 31982, Saudi Arabia; rhamad@kfu.edu.sa; 22 Central Laboratory, Theodor Bilharz Research Institute, Giza 12411, Egypt; 23 Department of Pharmacology, Faculty of Pharmacy, Delta University for Science and Technology, Gamasa 11152, Egypt

In the original publication [1], there was a mistake in Figure 2, as published. An image from group E was mistakenly placed in group C in Figure 2 due to an incorrect folder selection. This error was facilitated by the fact that the image selected for group C exhibited normal structures with no observable tissue changes, making it visually indistinguishable from the intended selection. The correct Figure 2 and it’s legend appears below. The authors state that the scientific conclusions are unaffected. This correction was approved by the Academic Editor. The original publication has also been updated.

The authors wish to replace the ‘Author Contributions’ statement for this article [1] with the following version since the wrong version was uploaded due to oversight:

**Author Contributions:** Conceptualization, S.S.; methodology, O.A.M., M.A.A.-R., L.A.S., M.M.S.A., J.A., M.I.E.A., A.A.F., A.A.J.A., W.F.B., A.A.H., S.A.-G., E.A.E., H.S.E.-w., A.S.D. and R.S.H.; software, S.S.; validation, O.A.M., M.A.A.-R. and S.S.; formal analysis, O.A.M. and S.S.; investigation, O.A.M., M.A.A.-R., L.A.S., M.M.S.A., J.A., M.I.E.A., A.A.F., A.A.J.A., W.F.B., A.A.H., S.A.-G., E.A.E., H.S.E.-w., A.S.D., R.S.H. and S.S.; resources, O.A.M., M.A.A.-R., L.A.S., M.M.S.A., J.A., M.I.E.A., A.A.F., A.A.J.A., W.F.B., A.A.H., S.A.-G., E.A.E., H.S.E.-w., A.S.D. and R.S.H.; software, S.S.; validation, O.A.M., M.A.A.-R. and S.S.; data curation, O.A.M., M.A.A.-R., L.A.S., A.A.F., A.A.J.A., A.A.H., E.A.E., H.S.E.-w. and A.S.D.; contributed to writing—original draft preparation, O.A.M., M.A.A.-R., L.A.S., M.M.S.A., J.A., M.I.E.A., A.A.F., A.A.J.A., W.F.B., A.A.H., S.A.-G., E.A.E., H.S.E.-w., A.S.D., R.S.H. and S.S.; writing—review and editing, O.A.M., M.A.A.-R., L.A.S., M.M.S.A., J.A., M.I.E.A., A.A.F., A.A.J.A., W.F.B., A.A.H., S.A.-G., E.A.E., H.S.E.-w., A.S.D., R.S.H. and S.S.; visualization, A.A.F., J.A., A.A.J.A., W.F.B. and A.A.H.; supervision, M.M.S.A., J.A., M.I.E.A., A.A.J.A., W.F.B., A.A.H., S.A.-G., A.S.D. and S.S.; project administration, O.A.M., A.S.D. and S.S.; funding acquisition, O.A.M. All authors have read and agreed to the published version of the manuscript.

The authors apologize for any inconvenience caused and state that the scientific conclusions are unaffected. The original article has been updated. This correction was approved by the Academic Editor. The original publication has also been updated.

## Figures and Tables

**Figure 2:**
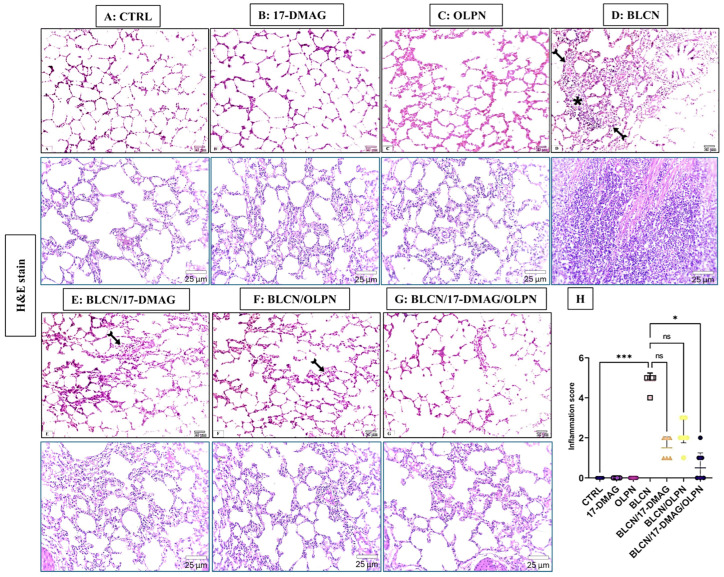
Effect of the alvespimycin, OLPN, and their combined therapy on the histological structure of the lung. Sections of CTRL (**A**), 17-DMAG (**B**), and OLPN (**C**), respectively, show normal lung architecture formed of thin-walled alveoli. Section of BLCN-treated rats (**D**) shows substantial inflammatory cellular infiltration (asterisk) and marked thickening of inter-alveolar septa (forked arrow) compared to CTRL, 17-DMAG, or OLPN rats. Lung sections from rats with pulmonary fibrosis treated with 17-DMAG (**E**), OLPN (**F**), and their combined therapy (**G**) reveal mild inflammatory cellular infiltration and alveolar septal thickening (forked arrows) as confirmed by the inflammation score (**H**). Significance is indicated by the pairwise comparisons. * *p* < 0.05 vs. BLCN; *** *p* < 0.001 vs. CTRL.
